# Why is end‐of‐life inpatient cost high among cancer patients? A prospective cohort study

**DOI:** 10.1002/cam4.7057

**Published:** 2024-03-08

**Authors:** Ishwarya Balasubramanian, Chetna Malhotra, the COMPASS study group

**Affiliations:** ^1^ Lien Centre for Palliative Care, Duke‐NUS Medical School Singapore Singapore; ^2^ Program in Health Services and Systems Research, Duke‐NUS Medical School Singapore Singapore

**Keywords:** aggressive treatment, cancer patients, end‐of‐life care, health care cost, Singapore, symptom burden

## Abstract

**Background:**

Inpatient cost for cancer patients is high during the last year of life, but reasons for this are not understood. We aim to understand the type of hospital admissions and inpatient services associated with an increase in inpatient cost in last year of life.

**Methods:**

We used survey and billing records of 439 deceased patients with a solid metastatic cancer, enrolled in a prospective cohort study. Based on cost per day of inpatient admissions, we classified admissions as low‐ or high‐intensity. We decomposed the inpatient cost into cost for different inpatient services. We examined the inpatient cost in the last year of life. We also assessed patient characteristics associated with higher inpatient cost in the next 3 months.

**Results:**

Towards death, proportion of inpatient cost for “maintenance care” increased while that for intensive care unit (ICU) and surgeries decreased. Low‐intensity, compared to high‐intensity admissions had a higher proportion of cost for “maintenance care” and a lower proportion for surgeries and ICU. Number of low‐intensity admissions increased more steeply towards death than high‐intensity admissions. Both admission types contributed equally to the share of inpatient cost. Older patients were less likely to have a high‐intensity admission (*β*:‐0.01, CI: −0.02, 0.00). Greater preference for life extension (*β*: 0.06, CI: 0.01, 0.11) and inaccurate prognostic belief were associated with higher cost of high‐intensity admissions (*β*: 0.32, CI: 0.03, 0.62).

**Conclusions:**

Findings suggest that inpatient costs in last year of life may be reduced if maintenance care is availed in low‐cost settings such as hospice/palliative care alongside steps to reduce non‐beneficial surgeries and ICU admissions.

## 1 BACKGROUND

Healthcare cost is high during the last months or end‐of‐life (EOL) among patients with cancer^1^, ^2^, ^3^ leading to significant financial burden for patients, families, and the healthcare system. This has been attributed mostly to high inpatient cost.^4^, ^5^ However, the big question is, “why does inpatient cost rise steeply at EOL?” Studies show that there is a high use of aggressive or non‐beneficial treatments at EOL, implying that these treatments drive up inpatient cost^2^, ^6^, ^7^ and that interventions to reduce these treatments may reduce EOL cost.^8^, ^9^ Others have argued that higher inpatient cost at EOL is due to inpatient services aimed at managing patients' symptoms,^10^, ^11^, ^12^ and that substantial reduction in cost savings may not be possible by reducing use of aggressive treatments.^13^, ^14^ Given these discrepant findings, it is imperative to understand what drives high inpatient cost at EOL.

Our first aim was to understand the type of hospital admissions and inpatient services associated with an increase in inpatient cost in the last year of life. Since aggressive treatments are costly, we used cost per day to classify type of hospital admissions into low‐ and high‐intensity admissions.^12^, ^15^ We validated this classification by testing whether ‘low‐intensity’ admissions have a lower proportion of inpatient cost incurred on aggressive treatments such as surgeries and intensive care unit (ICU) stays, and a higher proportion incurred on ‘maintenance care’ that is, care provided to manage patients' symptoms and function, including doctor review, feeding, pain management etc. Since pain and other symptoms are the chief causes of hospital admissions among patients with a metastatic cancer^16^, ^17^ and the prevalence of these symptoms increases as patients approach EOL,^18^ we hypothesized that the proportion of inpatient cost incurred on ‘maintenance care’, will increase, while that on surgeries and ICU stays will reduce at EOL, overall and within both high‐ and low‐intensity admissions.^12^ We also hypothesized that ‘low‐intensity’ admissions will increasingly constitute a higher proportion of inpatient cost as patients near death.

Our *second aim* was to assess patient characteristics associated with higher inpatient cost for high‐ and low‐intensity admissions over the next 3 months. Studies show that sicker patients are more likely to be admitted to the hospital, and to incur greater cost.^18^, ^19^, ^20^ We hence hypothesized that sicker patients will incur higher inpatient cost for both low‐ and high‐intensity admissions. Past literature also shows that younger age,^10^, ^21^, ^22^ greater preference for life extension,^4^, ^23^ inaccurate prognostic belief^24^ and having a private health insurance^2^, ^4^ are associated with use of aggressive interventions. We thus hypothesized that patients with these characteristics will incur higher inpatient cost for high‐intensity admissions.

## 2 METHODS

### 2.1 Setting

We conducted this study in Singapore, a high‐income Asian economy. Cancer is the leading cause of death in Singapore.^25^ Approximately 30% of health expenditure in Singapore is out‐of‐pocket, greater than the average for high‐income countries.^26^ A mandatory low‐cost public health insurance with high deductibles (Medishield Life) partially meets patients' healthcare cost. Individuals can top‐up this insurance coverage by purchasing a private health insurance.^27^

### 2.2 Study design and participants

We used data from COMPASS (Cost of Medical Care of Patients with Advanced Serious Illness in Singapore), a cohort of 600 patients diagnosed with a metastatic cancer. Following written consent, the study recruited patients (between July 2016 and March 2018) who met inclusion criteria for having a diagnosis of a solid metastatic cancer and, being 21 years or above, from two major public hospitals.

Participants were surveyed at baseline and every 3 months until they died and their billing data (starting January 2015) was obtained from hospital records. The study was approved by the Institutional Review Board at SingHealth. Details of the study protocol are published.^28^

### 2.3 Study variables

#### 2.3.1 Outcomes

##### Low and high intensity admissions

We calculated the cost per day for each admission (total gross cost/number of days in hospital) and the median cost per day, separately for admissions in private and subsidized wards. Admissions incurring lower than median cost per day in each type of ward were classified as low‐intensity admissions.

##### Total inpatient cost

We calculated total cost incurred in all inpatient admissions (gross cost before tax and subsidy), and separately for low‐ and high‐intensity admissions. The cost was adjusted for inflation to 2019 Singapore dollars (SGD) using the consumer price index from the Department of Statistics, Singapore.^29^

#### 2.3.2 Covariates

##### Age

Of patient during the baseline survey.

##### Symptom burden

At each survey, we assessed symptom burden using a composite measure of 10 items (shortness of breath, constipation, losing weight, vomiting, swelling, pain, dryness in mouth/throat, lack of energy, nausea, other symptoms); each item rated 0 (not at all) to 4 (very much). Total score calculated as sum of all items ranged from 0 to 40; a higher score indicated higher symptom burden.

##### Preference for life extension

We asked patients (at each survey) “If you had to make a choice now, would you prefer treatment that extends life as much as possible, or would you want treatment that cost you less?” Patients responded on a scale of 1–9, with 1 representing “extend life as much as possible at high cost” and 9 representing “no life extension at less cost”. We reverse coded it so that higher values indicate higher preference for life extension.

##### Prognostic belief

At each survey, patients were asked if they thought that the current treatment that they are taking would cure them (1 = inaccurate; 0 = correct/unsure).

##### Cancer site

Was categorized as breast, gynecologic/genitourinary, gastrointestinal, respiratory, and other.

### 2.4 Analysis

Using a sample of 439 deceased patients, we examined inpatient cost in the last year of life (Aim 1). To do this, we calculated the number of days between the date of each admission and death, and then the cost of all inpatient admissions within specified time intervals before death. If there were no admissions during the time interval, costs were considered to be zero. We decomposed the total inpatient cost (for all admissions, low‐ and high‐intensity admissions) into cost incurred for ward, investigations, surgery, maintenance, ICU and related units, procedures, consumables, drugs and others (Appendix S1). We examined the proportion of cost incurred on each inpatient service at 6–12, 0–6, 0–2 and 1 month before death. We plotted the mean total inpatient cost, total number of admissions and total number of days in hospital and the mean cost per day (total inpatient cost/total number of days in hospital) for each monthly interval over the last year of life, for all admissions, and low‐ and high‐intensity admissions separately.

To assess patient characteristics at survey n associated with higher inpatient spending over the next 3 months (Aim 2), our dependent variables were total inpatient cost, inpatient cost for low‐ and high‐intensity admissions, incurred by patients between date of survey n and n + 90 days. Independent variables included patients' symptom burden, age, preference for life extension and prognostic belief in survey n. The regression controlled for cancer site. As many patients incurred zero inpatient cost between two survey rounds, we ran two‐part models. The first part of the two‐part model used a logit function to identify patient characteristics associated with having nonzero inpatient cost. Conditional on cost being nonzero, the second part used a generalized linear model with log link and gamma distribution to identify patient characteristics associated with higher cost. To account for repeated observations for each patient, we used standard errors clustered at the patient level. Appendix S2 includes more details of the methods used. For patients who survived less than 90 days after survey n, the total cost in the next 90 days might be artificially lower, which might bias our results. We therefore conducted a sensitivity analysis in which we dropped patient‐time intervals where patient died within 30 days of survey n and estimated two‐part models using average monthly cost.

## 3 RESULTS

### 3.1 Sample characteristics

Of the 600 patients in our sample who completed at least one survey, 480 died during the study period. Our analytical sample constituted 439 deceased patients who had responded to at least one survey. The patients in our sample were on an average 61 [SD: 10.9] years, mostly Chinese (79%) and married (72%). Around 53% lived in 3‐ to 4‐room public housing, 41% had a primary education or less, and 62% had private health insurance (Table 1).

**TABLE 1:** Baseline sample characteristics (*N* = 439).

Characteristic	*N* (%)
Female	224 (51.0)
Age (range, 22–92), mean [SD], years	61 [10.9]
Chinese	347 (79.0)
Married	318 (72.4)
Highest educational attainment	
Primary and below	181 (41.2)
Secondary	136 (31.0)
Above secondary	122 (27.8)
Type of housing	
1–2 room public housing	31 (7.1)
3–4 room public housing	234 (53.3)
5 room public housing/private housing	174 (39.6)
Have a private health insurance	274 (62.4)
Preference for life extension/cost (range, 1–9), mean [SD]	4.4 [2.4]
Symptom Burden (range, 0–28), mean [SD]	5.5 [5.3]
Cancer site	
Breast	66 (15.0)
Colorectal	125 (28.5)
Gynecological/genitourinary	86 (19.6)
Respiratory	127 (28.9)
Others	35 (8.0)
Cost per day per admission (in SGD)	
Low‐intensity admission (range, 40–1511), mean [SD]	890 [219]
High‐intensity admission (range, 1176–26,735), mean [SD]	2611 [2446]

### 3.2 Relationship between admission intensity and inpatient services

Low‐intensity admissions had a higher proportion of inpatient cost incurred for ‘maintenance care’ and a lower proportion for ‘surgeries’ and ‘ICU’, compared to high‐intensity admissions. As patients approached death, proportion of inpatient cost in ‘maintenance care’ increased while that for ICU and surgery decreased within both low‐ and high‐intensity admissions. Despite this, the proportion of surgery and ICU cost in high‐intensity admissions remained high at about 10% of total inpatient cost in the last 2 months of life (Table 2). Actual cost of inpatient services is given in Table S1.

**TABLE 2:** Proportion of inpatient cost incurred on various inpatient services during the last year of life.

	Ward	Investigations	Surgery	Maintenance	Intensive care and related units	Procedures	Consumables	Drugs	Other
All admissions									
1 month before death	31.9	23.3	5.9	11.6	3.7	2.2	7.9	7.0	6.6
0–2 months before death	33.6	23.7	5.7	10.7	3.4	1.8	7.2	7.8	6.1
0–6 months before death	31.5	24.7	6.9	8.9	4.6	1.9	6.8	8.2	6.3
6–12 months before death	27.9	25.5	12.9	5.2	2.5	1.8	8.6	8.3	7.2
Low‐intensity admissions									
1 month before death	46.1	18.7	1.3	12.8	0.0	2.2	7.0	5.7	6.1
0–2 months before death	45.9	20.5	2.1	11.2	0.2	1.9	6.5	5.7	6.0
0–6 months before death	43.1	22.6	2.7	9.6	1.9	2.0	5.6	6.2	6.3
6–12 months before death	41.8	25.6	4.6	5.9	1.2	1.9	4.8	8.5	5.6
High‐intensity admissions									
1 month before death	18.3	27.6	10.3	10.5	7.2	2.1	8.7	8.2	7.0
0–2 months before death	19.0	27.6	9.9	10.2	7.2	1.7	8.1	10.2	6.1
0–6 months before death	19.6	26.9	11.2	8.3	7.4	1.8	8.1	10.4	6.4
6–12 months before death	17.0	25.5	19.4	4.6	3.6	1.8	11.6	8.2	8.5

### 3.3 Healthcare use and cost in the last year of life

Patients incurred a mean total inpatient cost of SGD 26860 in the last year of life, of which 71 percent was incurred in the last 6 months of life. The mean monthly total inpatient cost for the patients in our sample was SGD 2238 during the last year, which increased markedly during the last month of life (SGD5995).

**FIGURE 1:**
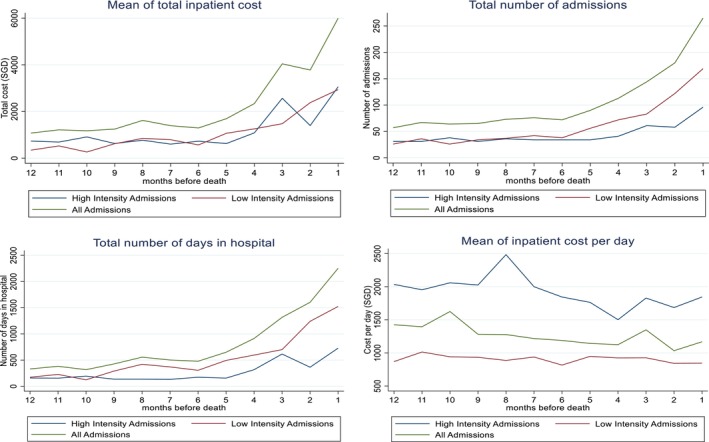
Trajectory of mean total inpatient cost, total admissions, total number of days in hospital and mean inpatient cost per day in the last year of life (All admissions, Low‐intensity admissions, High‐intensity admissions) *N* = 439.

Mean number of admissions and number of days in hospital increased markedly for both low‐ and high‐intensity admissions but the increase for low‐intensity admissions was steeper than that for high‐intensity admissions in last months of life. Relatedly, despite the low cost per day of low‐intensity admissions, the proportion of inpatient cost by low‐intensity admissions slightly surpassed that of high‐intensity admissions in the last 2 months of patients' life. Despite a fall in the proportion of inpatient cost by high‐intensity admissions, it remained high at 46% in the last 2 months before death due to the high cost per day for high‐intensity admissions. (Figure 1/Table S2).

### 3.4 Factors associated with cost of low‐ and high‐intensity admissions

Table 3 (Part 1) shows that patients with a higher symptom burden were more likely to experience an inpatient admission (*β*: 0.04, CI: 0.03, 0.05), low‐intensity admission (*β*: 0.04, CI: 0.03, 0.06) or high‐intensity admission (*β*: 0.02, CI: 0.01, 0.04). Older patients were less likely to experience any inpatient admission as well as high‐intensity admission (*β*:‐0.01, CI: −0.02,‐0.00). Greater preference for life extension (*β*: 0.06, CI: 0.01, 0.11) and having an inaccurate prognostic belief (*β*: 0.32, CI: 0.03, 0.62) were associated with higher cost of high‐intensity admissions. Greater preference for life extension was also associated with lower cost of low‐intensity admissions (*β*: −0.05, CI:−0.08, −0.02) (Table 3 Part 2). Having a private health insurance was associated with higher cost of low‐intensity admissions (*β*: 0.21, CI: 0.01, 0.42). The sensitivity analysis using average monthly cost is presented in Table S3. We dropped 28 patient‐time intervals in which death date was less than 30 days from survey n. The results of this model are qualitatively similar as above.

**TABLE 3:** Predictors of likelihood and cost of all, high and low‐intensity admissions.

	All Admissions		Low Intensity Admissions		High Intensity Admissions	
	β	[95% CI]	β	[95% CI]	β	[95% CI]
Part 1‐Logit						
Symptom burden score	0.04^c^	[0.03, 0.05]	0.04^c^	[0.03, 0.06]	0.02^c^	[0.01, 0.04]
Age	−0.01^b^	[−0.02, −0.00]	−0.01	[−0.02, 0.00]	‐0.01^a^	[−0.02, 0.00]
Preference for more life extension	0.00	[−0.04, 0.04]	‐0.01	[−0.06,0.04]	0.03	[−0.02, 0.08]
Inaccurate prognostic awareness	0.16	[−0.07, 0.38]	0.02	[−0.26, 0.30]	0.16	[−0.16, 0.47]
Have private health insurance	0.01	[−0.20, 0.22]	−0.05	[−0.32, 0.22]	0.18	[−0.09, 0.44]
Part 2‐generalized linear model						
Part 2‐GLM						
Symptom burden score	0.01	[−0.00, 0.02]	0.01^a^	[−0.00, 0.02]	0.00	[−0.01, 0.02]
Age	0.00	[−0.02, 0.01]	0.00	[−0.01, 0.01]	−0.01	[−0.02, 0.01]
Preference for more life extension	0.02	[−0.02, 0.06]	−0.05^c^	[−0.08, −0.02]	0.06^b^	[0.01, 0.11]
Inaccurate prognostic awareness	0.24^b^	[0.03, 0.46]	0.10	[−0.11, 0.30]	0.32^b^	[0.03, 0.62]
Have private health insurance	0.05	[−0.15, 0.25]	0.21^b^	[0.01, 0.42]	−0.15	[−0.44, 0.13]

^a^ *p* < 0.10.

^b^ *p* < 0.05.

^c^ *p* < 0.01.

## 4 DISCUSSION

In this study, we assessed why inpatient cost among patients with a metastatic cancer increased steeply towards the EOL. Although towards the EOL, the number of low‐intensity admissions increased more steeply than high‐intensity admissions, both type of admissions contributed equally to the share of inpatient cost due to the higher cost per day of high‐intensity admissions. Relatedly, proportion of inpatient cost due to maintenance care increased but surgery and ICU costs remained high at EOL. Results have implications for devising strategies to reduce inpatient costs at EOL.

Our results showed that mean number of admissions and days in hospital for low‐intensity admissions increased more steeply than high‐intensity admissions at EOL. As a result, despite lower cost per day, low‐intensity admissions constituted an increasingly higher share of overall inpatient cost as patients neared death. Relatedly, the proportion of inpatient cost spent in ‘maintenance care’ which constituted a large share of cost for low‐intensity admissions, increased at EOL. A previous study noted similar findings.^12^ This suggested that a high proportion of inpatient cost at EOL was consumed in managing sick patients.

That said, our results also showed that the mean number and number of days in hospital for high‐intensity admissions increased, albeit at a much slower rate than that of low‐intensity admissions resulting in the proportion of inpatient cost due to high‐intensity admissions decreasing significantly towards EOL. Despite this, it remained high at 46% in the last 2 months before death due to the high cost per day for high‐intensity admissions. Relatedly, despite the fall in the proportion of surgery and ICU cost in high‐intensity admissions, it was still high at 10% during the last 2 months before death suggesting that some patients do continue to receive aggressive treatments at EOL, as many previous studies have also suggested.^6^, ^30^, ^31^

Lastly, we found that patients with a higher symptom burden were more likely to experience both low‐ and high‐intensity admissions. This is consistent with literature showing that pain and other symptoms are the chief cause of hospital admissions among patients with a metastatic cancer.^16^, ^17^ Since the prevalence of these symptoms increases as patients approach EOL,^18^ it is not surprising that symptom burden is associated with more high‐ and low‐intensity admissions. We also found that patients with an inaccurate prognostic belief and a higher preference for life extension incurred higher cost for high‐intensity admissions. Previous studies have also shown that patients who believe that their treatments can cure them and those who have a higher preference for life extension are more likely to prefer and undergo life extending treatments,^4^, ^32^ and to incur higher healthcare cost. Notably, we found that younger patients were more likely to have a high‐intensity admission. This is consistent with studies showing that younger patients desired life‐prolonging care and were more likely to receive it.^33^ This may be because, conditional on being cured, younger people are expected to have a higher life expectancy than older people.

Our findings have policy implications. We recommend a two‐pronged approach to reduce EOL cancer cost. Firstly, inpatient cost due to hospital admissions involving maintenance care can potentially be reduced if very sick patients with a poor prognosis, at least those who do not prefer aggressive treatments, are shifted to “low‐cost” setting such as hospice. Despite the well‐established benefits of hospice care in reducing EOL cost while improving quality of life,^6^, ^34^, ^35^ its uptake remains low.^36^ Reasons include lack of awareness,^37^, ^38^, ^39^ stigma,^40^ misaligned financial incentives and difficulties initiating EOL discussions.^41^, ^42^, ^43^ Improving patient awareness and acceptance, oncologist training on when and how to refer, and creating appropriate financial incentives for referral, can increase hospice utilization and reduce low‐intensity admissions at EOL.

Secondly, high‐intensity admissions also constituted a large share of inpatient cost at EOL. We showed that inaccurate prognostic belief and greater preference for life extension were associated with a higher likelihood of high‐intensity admissions. These inaccurate prognostic beliefs can be addressed through effective communication with healthcare providers. However, more often, patients prognostic beliefs are driven by factors other than doctors communicated prognosis such as denial and hope.^44^ Changes in oncologists prescribing patterns (such as limiting life‐prolonging treatments on the basis of performance status), monitored‐care pathways,^45^ integration of palliative and oncology care^46^, ^47^ and payment models, such as partial capitated payments or case‐based reimbursements implemented with appropriate care quality measures,^48^ may reduce high‐intensity admissions.

The main strength of our study is that we merged administrative data with patient survey data, allowing us to assess patient characteristics such as prognostic belief and preference for life extension, that were associated with cost of inpatient admissions. At the same time, detailed records on inpatient services availed and their cost allowed us to decompose inpatient cost.

Our study has limitations. First, because we used survey data, our sample size is smaller compared to other studies relying solely on administrative data to evaluate healthcare cost among patients with a solid metastatic cancer. Second, we did not know the intent of surgeries performed; it is possible that palliation rather than life‐extension was the intent for some surgeries. Third, we did not have access to patients’ date of diagnosis for metastatic cancer. It is possible that some patients may have been diagnosed less than 1 year before death, and their zero costs reflected lack of disease/diagnosis rather than use of inpatient services. Fourth, we used a binary classification of admissions (low vs. high intensity) using a single indicator (cost per day) which could have led to some misclassification. Lastly, as no sampling frame was available to conduct a representative survey of patients with an advanced cancer, we adopted a non‐probability sampling approach to recruit patients for our study. This may have limited the generalizability of our results.

## 5 CONCLUSIONS

Increase in inpatient cost at EOL was driven by increase in the number of admissions and number of days in hospital, which were higher for low‐ than high‐intensity admissions. Due to the higher cost per day of high‐intensity admissions, both low and high‐intensity admissions contributed equally to the share of inpatient cost at EOL. Pattern of inpatient services shifted towards maintenance care towards the EOL but surgery and ICU cost were still high. Overall, our results suggest that many patients are admitted in inpatient setting for symptomatic/palliative treatment rather than life‐extending treatment. EOL cost of cancer may be reduced with integration of palliative and oncology care. Steps should be taken to ensure that maintenance care is availed in low‐cost settings such as hospices/palliative care alongside steps to reduce aggressive treatments.

## AUTHOR CONTRIBUTIONS

**Ishwarya Balasubramanian:** Conceptualization (equal); formal analysis (lead); methodology (equal); writing – original draft (lead); writing – review and editing (equal). **Chetna Malhotra:** Conceptualization (equal); formal analysis (supporting); methodology (equal); supervision (lead); writing – review and editing (equal).

## FUNDING INFORMATION

This work was supported by funding from Singapore Millennium Foundation (2015‐SMF‐0003) and Lien Centre for Palliative Care (LCPC‐IN14‐0003).

## CONFLICT OF INTEREST STATEMENT

The authors declare that they have no competing interests.

## ETHICS STATEMENT

The current study involves human subjects who provide their signed informed consent and the study was approved by the SingHealth Centralized Institutional Review Board (2015/2781). All methods were carried out in accordance with relevant guidelines and regulations.

## Supporting information


Data S1.

## Data Availability

Data is available on reasonable request from the corresponding author.
