# Supplementary material for: Why is end‐of‐life inpatient cost high among cancer patients? A prospective cohort study
Source: Cancer Med. 2024 Mar 8;13(4):e7057. doi: 10.1002/cam4.7057 (PMC10923043; doi:10.1002/cam4.7057)
Supplement: Supplementary file 1 — Data S1. [file CAM4-13-e7057-s001.docx]

**Appendix 1**

We decomposed the total inpatient cost (for all admissions, low- and high-intensity admissions) into cost incurred for the following inpatient services:

(a) *ward*: includes cost of stay in ward and daily treatment fee;

(b) *investigations*: includes cost of laboratory investigations, x-ray, scan, MRI and special investigations;

(c) *surgery*: includes surgeon fee, anesthetist fee, facility fee and cost of implants;

(d) *maintenance*: includes fee for doctor consultations, counselling, speech therapy, feeding, pain management, physiotherapy, occupational therapy, nutrition therapy etc.;

(e) *ICU and related units:* includes cost of stay in ICU/high dependency unit/cardiac care unit and daily treatment fee in these units;

(f) *procedures :*includes cost of procedures such as inpatient chemotherapy, radiotherapy, dialysis and some invasive procedures; does not include cost of procedures which are provided in outpatient setting

(g) *consumables*: includes cost of items used to treat patients such as bandages, IV solution, oxygen usage and other supplies from retail pharmacy; also includes cost of items and medical devices used in operation theatre

(h) *drugs*: includes cost of inpatient prescription drugs; does not include cost of outpatient prescription drugs

(i) *others*: includes cost of routine ward procedures such as dressing, non-treatment and miscellaneous cost.

**Appendix 2:**

For **Aim 1**, we analyse all inpatient admissions of 439 deceased patients in the last one year of life.

To do this, we started with a dataset where each inpatient admission was included as a separate row. For each inpatient admission, we calculated the length of stay using the admission and discharge dates. We also calculated the cost per day for each admission by dividing the total gross cost for the admission by length of stay. We then calculated the median cost per day for all admissions of all patients (separately for private and subsidized wards- median cost per day for private wards was S$1496 and for subsidized wards was S$ 1169). Each admission was classified as low- or high-intensity based on whether the cost per day for that admission was lower or higher than the median cost per day. Any patient could have both high and low-intensity admissions. From this dataset, we retained only the admissions during the last year of life corresponding to the 439 deceased patients. The months before death for each admission was calculated as the difference between deceased date for each patient and admission date for each admission.

Next, we restructured the above dataset at the level of **months before death.** To do this, we calculate the total gross cost incurred by each patient (in all, low- and high-intensity admissions) during every month in the last year of life. If there were no admissions in any month, cost was assumed to be zero. For each interval, the mean of total cost in all admissions (and low-intensity and high-intensity admissions) across 439 patients is calculated. The mean total cost for all admissions was equal to the mean total cost for low- and high-intensity admissions. This was plotted in Figure 1a. We also plotted the total number of admissions and total number of days in hospital during each time interval for all, low- and high-intensity admissions (Figures 1b and 1c). Lastly, we calculated the mean inpatient cost per day for each interval as the total cost divided by the number of days in hospital of all the admissions in that interval (Figure 1d).

For **Aim 2**, we examined patient characteristics at survey n that were associated with cost of inpatient admission in the next 90 days. The regression analysis (Table 3) included all admissions for the 439 decedents that happened after their baseline survey till 3 months after their last survey.

To do this, we first input the survey dates for each patient. Next, we assign survey n for an admission if the admission date falls within 90 days of survey n. In a few cases where the time between two surveys was less than 90 days and the admission happened within 90 days of survey n but after survey n+1, we still assign survey n to the admission. Admissions before baseline survey and those that happened more than 90 days from a survey were dropped. Finally, we aggregated this dataset at the level of PT-survey wave. For every patient and every survey wave n answered by the patient, the cost of all (and low- and high-intensity) admissions that were assigned survey n (admissions that happened 90 days after date of survey n) are added. If a patient has answered, say three surveys, but has cost data only for one survey, we assume zero cost for the other two survey waves the patient has answered. This is the dependent variable for the regression analysis. The patient characteristics of survey n are the independent variables for the regression analysis.

**Supplementary Tables:**

**Supplementary Table 1: Average total inpatient cost incurred for different inpatient services during the last year of life**

|  | Ward | Investigations | Surgery | Maintenance | Intensive Care and related units | Procedures | Consumables | Drugs | Other | Average total inpatient cost |
| --- | --- | --- | --- | --- | --- | --- | --- | --- | --- | --- |
| **All Admissions** |  |  |  |  |  |  |  |  |  |  |
| 1 month before death | 1915.3 | 1396.1 | 354.1 | 695.1 | 218.9 | 129.0 | 471.6 | 419.4 | 395.8 | 5995.2 |
| 0-2 months before death | 3286.1 | 2317.5 | 553.2 | 1050.0 | 331.0 | 175.1 | 707.9 | 758.2 | 593.2 | 9771.9 |
| 0-6 months before death | 6033.5 | 4734.7 | 1323.2 | 1713.1 | 883.8 | 360.8 | 1305.9 | 1576.3 | 1210.5 | 19141.4 |
| 6-12 months before death | 2154.1 | 1971.3 | 992.6 | 398.0 | 196.1 | 142.4 | 663.8 | 643.9 | 556.1 | 7718.4 |
| **Low-Intensity Admissions** |  |  |  |  |  |  |  |  |  |  |
| 1 month before death | 1355.0 | 550.3 | 38.8 | 375.0 | 0.0 | 64.4 | 205.0 | 167.6 | 180.2 | 2936.2 |
| 0-2 months before death | 2440.9 | 1087.7 | 109.9 | 595.0 | 11.2 | 100.8 | 345.4 | 304.7 | 320.4 | 5315.8 |
| 0-6 months before death | 4176.4 | 2192.4 | 263.0 | 926.2 | 187.1 | 191.5 | 543.2 | 596.6 | 605.6 | 9681.8 |
| 6-12 months before death | 1418.0 | 867.6 | 154.6 | 200.9 | 42.3 | 64.3 | 162.9 | 288.7 | 189.2 | 3388.4 |
| **High-Intensity Admissions** |  |  |  |  |  |  |  |  |  |  |
| 1 month before death | 560.3 | 845.8 | 315.4 | 320.1 | 218.9 | 64.5 | 266.6 | 251.8 | 215.5 | 3059.0 |
| 0-2 months before death | 845.2 | 1229.8 | 443.3 | 455.0 | 319.8 | 74.4 | 362.5 | 453.4 | 272.8 | 4456.2 |
| 0-6 months before death | 1857.1 | 2542.3 | 1060.2 | 786.9 | 696.7 | 169.2 | 762.7 | 979.7 | 604.9 | 9459.6 |
| 6-12 months before death | 736.0 | 1103.7 | 838.1 | 197.1 | 153.8 | 78.1 | 501.0 | 355.2 | 367.0 | 4330.0 |

**Supplementary Table 2: Mean total inpatient cost, total number of days in hospital, total number of admissions and mean inpatient cost per day during the last year of life**

| Months before death | Mean total inpatient cost (SGD) | | | Total days in hospital | | |
| --- | --- | --- | --- | --- | --- | --- |
|  | All | Low Intensity | High Intensity | All | Low Intensity | High Intensity |
| 0-1 | 5995 | 2936 | 3059 | 2252 | 1524 | 728 |
| 1-2 | 3777 | 2380 | 1397 | 1604 | 1240 | 364 |
| 2-3 | 4043 | 1477 | 2566 | 1316 | 700 | 616 |
| 3-4 | 2339 | 1255 | 1084 | 913 | 596 | 317 |
| 4-5 | 1693 | 1067 | 627 | 650 | 494 | 156 |
| 5-6 | 1294 | 567 | 727 | 478 | 305 | 173 |
| 6-7 | 1392 | 791 | 601 | 502 | 370 | 132 |
| 7-8 | 1618 | 848 | 769 | 556 | 420 | 136 |
| 8-9 | 1246 | 619 | 628 | 427 | 291 | 136 |
| 9-10 | 1173 | 264 | 910 | 317 | 123 | 194 |
| 10-11 | 1214 | 524 | 690 | 382 | 227 | 155 |
| 11-12 | 1075 | 343 | 732 | 331 | 173 | 158 |
| Months before death | Total number of admissions | | | Mean inpatient cost per day (SGD) | | |
|  | All | Low Intensity | High Intensity | All | Low Intensity | High Intensity |
| 0-1 | 265 | 169 | 96 | 1169 | 846 | 1845 |
| 1-2 | 180 | 122 | 58 | 1034 | 842 | 1685 |
| 2-3 | 144 | 83 | 61 | 1349 | 926 | 1829 |
| 3-4 | 113 | 72 | 41 | 1125 | 924 | 1502 |
| 4-5 | 90 | 56 | 34 | 1144 | 948 | 1763 |
| 5-6 | 72 | 38 | 34 | 1189 | 817 | 1844 |
| 6-7 | 76 | 42 | 34 | 1217 | 938 | 2000 |
| 7-8 | 73 | 37 | 36 | 1277 | 887 | 2484 |
| 8-9 | 65 | 34 | 31 | 1282 | 934 | 2026 |
| 9-10 | 64 | 26 | 38 | 1625 | 942 | 2059 |
| 10-11 | 67 | 36 | 31 | 1395 | 1014 | 1953 |
| 11-12 | 57 | 26 | 31 | 1426 | 869 | 2035 |

**Supplementary Table 3: Predictors of likelihood and average monthly cost of all, high and low-intensity admissions**

|  | **All Admissions** | | **Low Intensity Admissions** | | **High Intensity Admissions** | |
| --- | --- | --- | --- | --- | --- | --- |
|  | **β** | **[95% CI]** | **β** | **[95% CI]** | **β** | **[95% CI]** |
| **Part 1-Logit** |  |  |  |  |  |  |
| Symptom burden score | 0.04*** | [0.03,0.05] | 0.04*** | [0.03,0.06] | 0.02*** | [0.01,0.04] |
| Age | -0.01** | [-0.02,-0.00] | -0.01 | [-0.02,0.00] | -0.01* | [-0.02,0.00] |
| Preference for more life extension | 0.00 | [-0.04,0.04] | -0.01 | [-0.06,0.04] | 0.03 | [-0.02,0.08] |
| Inaccurate prognostic awareness | 0.13 | [-0.09,0.36] | 0.02 | [-0.26,0.30] | 0.16 | [-0.16,0.47] |
| Have private health insurance | 0.01 | [-0.20,0.22] | -0.05 | [-0.32,0.22] | 0.18 | [-0.09,0.44] |
| **Part 2-Generalized linear model** |  |  |  |  |  |  |
| Symptom burden score | 0.01 | [-0.00,0.02] | 0.01* | [-0.00,0.02] | 0.00 | [-0.01,0.02] |
| Age | 0.00 | [-0.01,0.01] | 0.00 | [-0.01,0.01] | 0.00 | [-0.02,0.01] |
| Preference for more life extension | 0.02 | [-0.02,0.06] | -0.05*** | [-0.09,-0.01] | 0.06** | [0.01,0.12] |
| Inaccurate prognostic awareness | 0.26** | [0.04,0.47] | 0.08 | [-0.15,0.31] | 0.36** | [0.07,0.65] |
| Have private health insurance | 0.09 | [-0.11,0.29] | 0.20* | [-0.01,0.41] | -0.10 | [-0.38,0.19] |

* p<0.10, ** p<0.05, *** p<0.01
